# Greater Food Reward Sensitivity Is Associated with More Frequent Intake of Discretionary Foods in a Nationally Representative Sample of Young Adults

**DOI:** 10.3389/fnut.2016.00033

**Published:** 2016-08-18

**Authors:** Tonja R. Nansel, Leah M. Lipsky, Miriam H. Eisenberg, Denise L. Haynie, Danping Liu, Bruce Simons-Morton

**Affiliations:** ^1^Health Behavior Branch, Division of Intramural Population Health Research, Eunice Kennedy Shriver National Institute of Child Health and Human Development, Bethesda, MD, USA; ^2^Biostatistics and Bioinformatics Branch, Division of Intramural Population Health Research, Eunice Kennedy Shriver National Institute of Child Health and Human Development, Bethesda, MD, USA

**Keywords:** food reward sensitivity, food reward responsivity, dietary intake, eating behaviors, young adults

## Abstract

Food reward sensitivity may influence individual susceptibility to an environment replete with highly palatable foods of minimal nutritional value. These foods contain combinations of added sugar, fat, and/or salt that may enhance their motivational salience. This study examined associations of food reward sensitivity with eating behaviors in the NEXT Generation Health Study, a nationally representative sample of U.S. young adults. Participants (*n* = 2202) completed self-report measures including the Power of Food Scale, assessing food reward sensitivity, and intake frequency of 14 food groups. Multiple linear regressions estimated associations of food reward sensitivity with each of the eating behaviors adjusting for covariates. Higher food reward sensitivity was associated with more frequent intake of fast food (*b* ± linearized SE = 0.24 ± 0.05, *p* < 0.001), sweet and salty snacks (0.21 ± 0.05, *p* < 0.001), foods made with cheese (0.14 ± 0.06, *p* = 0.03), soda (0.12 ± 0.04, *p* = 0.009), processed meats (0.12 ± 0.05, *p* = 0.045), and fish (0.08 ± 0.03, *p* = 0.03) but was not associated with intake frequency of fruit or juice, green or orange vegetables, beans, whole grains, nuts/seeds, or dairy products. Food reward sensitivity was associated with greater intake of discretionary foods but was not associated with intake of most health-promoting foods, suggesting food reward sensitivity may lead to preferential intake of unhealthful foods.

## Introduction

Poor diet is the largest contributor to early death globally (1) and is linked to numerous adverse health outcomes independent of body mass index (BMI) (2–6). In the U.S., population dietary intake is characterized by inadequate intake of fruit, vegetables, whole grains, and excess intake of discretionary foods (those of minimal nutritional value) such as cakes, cookies, pastries, ice cream, chips/crisps, and fried foods (7). Across developed countries, diets are characterized by excessive intake of unhealthful foods (8), sugar (9), and salt (10) – the latter two of which are primary ingredients in many discretionary foods. These foods are ubiquitous (11) and highly marketed, especially to youth (12, 13), and many people experience difficulty moderating their intake (14, 15).

Emerging evidence suggests that food reward sensitivity, the neurologic reward response to food stimuli, may explain variations in susceptibility to highly palatable foods in the environment (14, 16–19). Such foods are characteristically energy dense and nutrient-poor, typically containing added sugar, fat, and/or salt in combinations that maximize palatability (20–23). An emerging hypothesis is that the highly processed nature of these foods enhances their motivational salience due to high caloric density, elevated potency, quicker absorption, and addition of flavor enhancers (14). Additionally, these high calorie foods stimulate greater activation of brain reward circuitry relative to low calorie foods (24), potentially contributing to their consumption for hedonic reasons, rather than homeostatic needs.

The Power of Food Scale (PFS) was developed as a measure of individual differences in appetitive responsiveness to the rewarding properties of food in the environment (17, 25, 26) and has been used as a measure of food reward sensitivity [e.g., Ref. (27–29)]. Higher scores on the PFS were associated with greater intensity of magnetoencephalography response to food stimuli (30, 31), greater connectivity in the visual cortex during imaging of food cues (32), and greater shifts in brain networks paralleling those observed with addictive behaviors in response to food cues (33). Higher food reward sensitivity as measured by the PFS was associated with greater reported food cravings (25, 32, 34, 35) and greater attentional bias toward high calorie food pictures (35). Additionally, persons scoring higher on the PFS reported greater food cravings in response to food stimuli than those scoring lower on the PFS (32, 33). PFS scores were associated with BMI in clinical weight loss and bariatric surgery patients (26, 28, 29, 36); however, findings in non-clinical samples are inconsistent (25, 26, 37, 38).

Few studies have examined the association of food reward sensitivity with dietary intake and eating behaviors. In experimental paradigms, greater food reward sensitivity as assessed with the PFS was associated with greater likelihood of consuming chocolate against experimenter instructions (34), greater likelihood of choosing an unhealthy snack versus healthy snack or non-snack item (35), and greater food intake (27, 39). In the latter two studies, findings were conflicting as to whether food reward sensitivity was associated only with intake of highly palatable food (39) versus total intake of both highly palatable and bland food (27). Whether the association of dietary intake with food reward sensitivity differs according to food group is a critical knowledge gap. If higher food reward sensitivity increases intake due to hedonic eating (eating for pleasure in the absence of energetic need), this suggests that such non-homeostatic eating behavior would most likely target foods with the greatest hedonic value. Additional research elucidating influences on individual susceptibility to the effect of exposure to food cues on eating behaviors may inform the development of interventions targeting improved diet quality. To our knowledge, previous research has not examined the association of the PFS with eating behaviors in a free-living sample or examined how PFS is related to intake of different food groups.

The purpose of this study was to examine the association of food reward sensitivity, as measured by the PFS, with reported frequency of intake of a range of healthful and unhealthful food groups in a nationally representative sample of young adults. We hypothesized that greater food reward sensitivity would be associated with greater intake of discretionary food groups but would not be associated with intake of more healthful food groups.

## Materials and Methods

### Design and Participants

Data come from wave 5 (2 years after high school) of the NEXT Generation Health Study, a longitudinal, prospective study of a nationally representative cohort of U.S. adolescents enrolled in 10th grade during the 2009–2010 school year and assessed in annual waves. Primary sampling units were school districts or groups of school districts stratified across the nine U.S. Census divisions; out of the 137 schools randomly selected, 81 (59%) agreed to participate. Classrooms of 10th graders within each of these schools were randomly selected to participate. Schools with large percentages of African-American students were oversampled to provide reliable estimates for this subgroup; a sufficient number of Hispanic students were obtained to provide reliable subgroup estimates without oversampling. Participants completed self-administered surveys online. The study protocol was approved by the Institutional Review Board of the Eunice Kennedy Shriver National Institute of Child Health and Human Development; participants provided written informed consent. Out of the 3,796 students originally invited to participate, 2,785 consented and participated and 2,202 were assessed in wave 5.

### Measures

#### Outcome

Intake frequency of food groups was assessed using items modified from the Youth Risk Behavior Surveillance System (40) and the multinational Health Behavior in School-aged Children study (41). Each item queried, “During the past 7 days, how many times did you eat or drink…?” Food groups assessed included 100% fruit juice, fruit, green vegetables, orange vegetables, beans, whole grain foods, soda/pop (not including diet), sweet or salty snacks, nuts or seeds, processed meats, fish, dairy products, and foods made with cheese (e.g., quesadillas, lasagna). Responses ranged from never to four or more times per day. Additionally, intake frequency of fast food was assessed with an item querying “How often did you eat food from a fast food restaurant (for example, McDonalds, KFC, Pizza Hut, and Taco Bell)?” Responses ranged from never to five or more days per week.

#### Variable of Interest

Food reward sensitivity was assessed in wave 5 using the 15-item PFS. Each item is rated on a 5-point Likert Scale. Items query responses to food being available in the environment (Food Available subscale, e.g., “When I know a delicious food is available, I can’t help myself from thinking about having some”), response to food being directly present (Food Present subscale, e.g., “If I see or smell a food I like, I get a powerful urge to have some”), and response to the taste of food (Food Tasted subscale, e.g., “I love the taste of certain foods so much that I can’t avoid eating them even if they’re bad for me”). Cronbach’s alpha of the aggregate score in the current sample was 0.94. In previous studies, the measure has demonstrated strong internal consistency (Cronbach’s alpha = 0.91), test–retest reliability (*r* = 0.77, *p* < 0.001) (25, 26), and has shown associations with brain activity in response to viewing images of food versus control images (30, 32).

#### Covariates

Baseline self-reported sex, age, ethnicity (Hispanic or Latino; not Hispanic or Latino), race, parent education, family affluence, year five self-reported height, BMI, physical activity, and smoking were all selected as covariates *a priori*. Participants reported race using predefined categories (Black or African-American, White, Asian, American Indian or Alaska Native, Native Hawaiian or other Pacific Islander). Responses were categorized as White, African-American, Hispanic, and Other. Participant responses regarding household car and computer ownership, family vacations, and bedroom sharing were used to calculate the previously validated Family Affluence Scale (42); scores range from 0 (low affluence) to 7 (high affluence). Highest parent education, ascertained during the consent process, was categorized as less than high school graduate/high school graduate/some college/bachelor’s degree/graduate degree. Participants self-reported their height, weight, number of hours of past-week vigorous physical activity (“enough to get out of breath or sweat”), and number of cigarettes smoked per day.

### Analyses

Multiple imputation by chained equations, assuming missing-at-random (43) was used to deal with item non-response. The algorithm iteratively imputes missing variables by estimating its distribution conditional on other variables. Fifty imputed datasets were generated using IVEware (44). Each dataset was analyzed separately, and the results were combined using Rubin’s rule in StataSE version 14 (College Station, TX, USA). Participant characteristics were summarized with means and SE for continuous variables and percentages for categorical variables. Multiple linear regression estimated associations of the PFS aggregate score with intake frequency of each of the eating behaviors adjusting for sex, age, race/ethnicity, family affluence, parent education, height, BMI, and physical activity. *Post hoc*, we also examined the association of each of the three PFS subscales (Food Available, Food Present, and Food Tasted) with intake frequency of each of the eating behaviors, including the same covariates. Survey estimation methods were used to account for the complex sampling design. The regression coefficient of PFS is interpreted as the mean increase in the frequency of eating behavior (times per day or days per week) per unit increase in PFS.

## Results

Sample characteristics are shown in Table 1. The mean age of the sample was 20 years; 59% were females. Food groups showing the highest intake frequency were dairy products, foods made with cheese, fruit, and whole grain foods. Foods consumed least frequently were fish, beans, and orange vegetables.

**Table 1:** **Weighted sample characteristics of the NEXT Generation Health Study at wave 5**.

	Mean or % ± SE
**Participant characteristics**	
Age (years)	20.27 ± 0.23
Sex	
Male	40.78 ± 1.85
Female	59.22 ± 1.85
Race/ethnicity	
Non-hispanic white	60.70 ± 5.36
Non-hispanic black	13.63 ± 3.36
Hispanic	20.25 ± 3.88
Other	5.43 ± 1.04
Parent education	
<High school	8.05 ± 2.17
High school graduate	25.05 ± 1.90
Some college	37.90 ± 2.08
Bachelor’s degree	16.06 ± 1.85
Graduate degree	12.94 ± 2.18
Family affluence scale	5.47 ± 0.10
Vigorous physical activity (hours per week)	2.61 ± 0.12
Body mass index	25.67 ± 0.33
Power of Food Scale	2.06 ± 0.02
**Food group intake frequency^a^**	
100% fruit juice	0.74 ± 0.04
Fruit	0.93 ± 0.04
Green vegetables	0.86 ± 0.04
Orange vegetables	0.50 ± 0.03
Beans	0.45 ± 0.03
Whole grain foods	0.94 ± 0.04
Nuts or seeds	0.58 ± 0.03
Fish	0.38 ± 0.03
Dairy products	0.94 ± 0.04
Soda/pop (not diet)	0.76 ± 0.04
Processed meats	0.76 ± 0.04
Foods made with cheese (e.g., quesadillas and lasagna)	0.94 ± 0.06
Sweet or salty snacks	0.85 ± 0.03
Fast food	1.13 ± 0.06

*^a^Values indicate frequency of intake per day, with the exception of fast food, which indicates frequency of intake per week*.

Higher food reward sensitivity, as measured by the PFS aggregate score, was associated with more frequent intake of fast food (*b* ± linearized SE = 0.24 ± 0.05, *p* < 0.001), sweet/salty snacks (0.21 ± 0.05, *p* < 0.001), foods made with cheese (0.14 ± 0.06, *p* = 0.03), soda (0.12 ± 0.04, *p* = 0.009), processed meats (0.12 ± 0.05, *p* = 0.045), and fish (0.08 ± 0.03, *p* = 0.03) (Table 2). The PFS aggregate score was not associated with intake frequency of fruit or fruit juice, green or orange vegetables, beans, whole grains, nuts/seeds, or dairy products.

**Table 2:** **Linear regression models estimating associations of food reward responsivity with intake frequency**.

	Power of Food Scale Aggregate Score		Power of food subscales					

			Food available		Food present		Food tasted	
				
Food group^a^	*b* ± SE	*p*	*b* ± SE	*p*	*b* ± SE	*p*	*b* ± SE	*p*
100% fruit juice	−0.003 ± 0.03	0.94	−0.01 ± 0.03	0.65	−0.03 ± 0.03	0.21	0.05 ± 0.04	0.24
Fruit	0.05 ± 0.04	0.21	0.03 ± 0.03	0.41	−0.002 ± 0.03	0.94	0.10 ± 0.04	0.03
Green vegetables	0.07 ± 0.04	0.10	0.06 ± 0.04	0.10	0.02 ± 0.03	0.61	0.09 ± 0.04	0.03
Orange vegetables	0.03 ± 0.03	0.29	0.04 ± 0.03	0.21	−0.01 ± 0.02	0.55	0.06 ± 0.03	0.11
Beans	0.03 ± 0.03	0.33	0.06 ± 0.03	0.07	−0.009 ± 0.02	0.70	0.03 ± 0.03	0.24
Whole grain foods	0.04 ± 0.04	0.36	0.05 ± 0.04	0.21	−0.002 ± 0.03	0.96	0.06 ± 0.04	0.18
Nuts or seeds	0.06 ± 0.03	0.07	0.08 ± 0.03	0.01	0.02 ± 0.03	0.43	0.04 ± 0.02	0.12
Fish	0.08 ± 0.03	0.03	0.10 ± 0.03	0.01	0.04 ± 0.03	0.17	0.05 ± 0.03	0.13
Dairy products	0.09 ± 0.05	0.07	0.07 ± 0.04	0.09	0.07 ± 0.04	0.12	0.08 ± 0.04	0.06
Soda/pop (not diet)	0.12 ± 0.04	0.009	0.10 ± 0.04	0.18	0.10 ± 0.03	0.01	0.09 ± 0.04	0.047
Processed meats	0.12 ± 0.05	0.045	0.11 ± 0.05	0.07	0.09 ± 0.04	0.03	0.08 ± 0.05	0.08
Foods made with cheese (e.g., quesadillas, lasagna)	0.14 ± 0.06	0.03	0.12 ± 0.05	0.04	0.12 ± 0.05	0.02	0.10 ± 0.06	0.09
Sweet or salty snacks	0.21 ± 0.05	<0.001	0.18 ± 0.04	0.001	0.18 ± 0.04	<0.001	0.14 ± 0.04	0.002
Fast food	0.24 ± 0.05	<0.001	0.23 ± 0.05	<0.001	0.19 ± 0.04	<0.001	0.17 ± 0.05	0.002

*^a^Responses for food groups other than fast food indicate frequency of intake per day; responses for fast food indicate frequency of intake per week. Models were adjusted for sex, age, race/ethnicity, family affluence, parent education, height, body mass index, and vigorous physical activity*.

The association of PFS subscales with intake frequency was consistent across subscales for sweet/salty snacks and fast food (Table 2). While associations of the PFS subscales with soda, processed meats, and foods made with cheese were similar in magnitude, they were not statistically significant across all subscales. The Food Present subscale yielded associations most consistent with those of the aggregate score; the Food Tasted subscale yielded the least consistent associations.

## Discussion

In this nationally representative sample of young adults, higher food reward sensitivity as measured by the PFS aggregate score was associated with more frequent intake of discretionary food categories including sweet and salty snacks, processed meat, cheesy foods, and fast food. However, except for fish, food reward sensitivity was not associated with intake frequency of more healthful foods including fruit, vegetables, beans, whole grains, nuts/seeds, and dairy products. This pattern implies an association of food reward sensitivity with intake primarily of the foods with the least nutritional value. This selective association is consistent with a body of literature indicating that the foods most likely to induce hedonic overeating are highly processed and energy dense, containing added sugar, fat, and salt (14, 15, 23, 45). These foods are difficult to resist for many (15), are often consumed despite a desire or intention to reduce their intake (14, 15), and are overconsumed throughout the population (46, 47). In two previous studies, scores on the PFS were associated with other dietary variables representing aspects or degrees of uncontrolled eating, including emotional eating, external eating, disinhibition, and binge eating (25, 48); further investigation is needed to understand how food reward sensitivity as measured by the PFS relates to other determinants of eating behavior. Understanding contributors to increased intake of nutrient-poor foods is of public health concern not only because their intake promotes excess weight but also because it is associated with poorer health independent of weight status (1, 2, 4).

The Food Tasted subscale of the PFS showed the least consistent associations with eating behaviors of the three subscales, echoing previous research showing lesser utility of the Food Tasted subscale versus the Food Present and Food Available subscales (36, 38, 48). Research examining the hedonic value of food indicates a distinction between taste perception and reward value. The taste appeal of a food (liking) and the desire to consume a food (wanting) are believed to have independent neural pathways (49), with the latter being a stronger driver of excess intake (50). While further investigation is indicated, this body of research suggests that the association of PFS with increased intake of highly palatable foods might not be attributable simply to the pleasant taste of these foods. The taste of a highly palatable food is likely to be rated highly but variation in food reward sensitivity may further impact the degree of one’s desire to consume it. This supposition is further supported by an animal model study in which the reinforcing value of food was dependent not on sweet taste but only on the actual presence of sugar in the food (51), which strongly activates brain reward regions (52).

Findings, herein, suggest that food reward sensitivity may be a relevant mechanism by which individual susceptibility interacts with the food environment to adversely impact dietary behavior. The lack of a consistent association of food reward sensitivity with more nutrient-rich food groups suggests that individual differences in food reward sensitivity may have minimal effect on intake in an environment characterized primarily by these food groups. However, as highly processed, highly palatable foods have become ubiquitous and normative, food reward sensitivity may play an important role in influencing dietary intake through a number of potential mechanisms. For example, high food reward sensitivity could impact eating *via* increased attention to environmental food cues (53), increased mental elaboration in response to food cues (e.g., thoughts about the hedonic properties of the food) (53), and subsequent inhibition of goals regarding healthful eating (54). When attempting to regulate dietary intake of highly palatable foods, effortful resistance results in a depletion of cognitive and emotional energy (55); consequently, efforts to restrict intake often fail. If indeed food reward sensitivity reflects an increased vulnerability to an obesogenic environment, efforts to modify the environment may be more successful than efforts to increase self-control.

A notable strength of this study is the use of a large, contemporary, nationally representative sample. However, several limitations should be considered when interpreting these findings. The self-report dietary screener is more susceptible to measurement error than more comprehensive methods (56) but is considered adequate for population-level surveillance of eating behaviors (57). Because the measure indicates intake frequency rather than amount, it is not possible to determine whether the increased intake frequency of discretionary foods observed among those with higher food reward sensitivity resulted in greater overall energy intake. Additionally, the study is unable to examine cross-cultural or cross-national differences in regards to the association of food reward sensitivity with dietary intake. It is important to note that the body of research using the PFS is relatively small, and the construct it measures is not yet fully understood, limiting the ability to compare these findings to other epidemiological studies.

A substantial proportion of the diets of both youth and adults in the U.S. is from discretionary foods high in energy and low in other nutrients (46, 47), adversely impacting public health (1). Difficulty regulating intake of these foods is common (14, 15). Findings from this study suggest that food reward responsivity, understood as the neurologic reward response to food stimuli, may influence individual susceptibility to these foods. However, research on these constructs is in its early stages, and findings must be treated as preliminary. Further research examining the association of food reward sensitivity with dietary intake using more precise measures is needed, as is research examining the interplay of food reward sensitivity with other relevant demographic, behavioral, and environmental variables. Additional work will be needed to determine how to most effectively use resulting advances toward the development of approaches to more effectively improve dietary behavior; however, findings lend support to the importance of environmental and policy approaches to decrease the widespread availability of highly palatable, nutrient-poor foods.

## Author Contributions

TN conceptualized the research question, interpreted findings, and drafted and edited the manuscript. LL and DL analyzed data and contributed to interpretation of findings. ME contributed to drafting of the manuscript. LL, ME, DL, DH, and BS-M contributed to editing of the manuscript. BS-M and DH directed the design and implementation of the study from which these data were drawn. All authors read and approved the final manuscript.

## Conflict of Interest Statement

The authors declare that the research was conducted in the absence of any commercial or financial relationships that could be construed as a potential conflict of interest.
